# Inhibition of thrombin on endothelium enhances recruitment of regulatory T cells during IRI and when combined with adoptive Treg transfer, significantly protects against acute tissue injury and prolongs allograft survival

**DOI:** 10.3389/fimmu.2022.980462

**Published:** 2023-01-30

**Authors:** Qi Peng, Anna Nowocin, Kulachelvy Ratnasothy, Richard A. Smith, Lesley A. Smyth, Robert I. Lechler, Anthony Dorling, Giovanna Lombardi

**Affiliations:** ^1^ Centre for Nephrology, Urology and Transplantation, School of Immunology and Mucosal Biology, King’s College London, London, United Kingdom; ^2^ School of Health, Sport and Bioscience, University of East London, London, United Kingdom

**Keywords:** murine heart transplantation, murine renal ischemia/reperfusion injury, coagulation cascade, thrombin, membrane-localizing thrombin inhibitor PTL060, regulatory T cells

## Abstract

Ischemia-reperfusion injury (IRI) amplifies T cell alloimmune responses after transplantation with thrombin playing a key pro-inflammatory role. To explore the influence of thrombin on regulatory T cell recruitment and efficacy we used a well-established model of IRI in the native murine kidney. Administration of the cytotopic thrombin inhibitor PTL060 inhibited IRI, and by skewing expression of chemokines (reducing CCL2 and CCL3 but increasing CCL17 and CCL22) increased the infiltration of M2 macrophages and Tregs. When PTL060 was combined with infusion of additional Tregs, these effects were further amplified. To test the benefits of thrombin inhibition in a transplant model, BALB/c hearts were transplanted into B6 mice with or without perfusion with PTL060 in combination with Tregs. Thrombin inhibition or Treg infusion alone led to small increments in allograft survival. However, the combined therapy led to modest graft prolongation by the same mechanisms as in renal IRI; graft survival was accompanied by increased numbers of Tregs and anti-inflammatory macrophages, and reduced expression of pro-inflammatory cytokines. While the grafts succumbed to rejection associated with the emergence of alloantibody, these data suggest that thrombin inhibition within the transplant vasculature enhances the efficacy of Treg infusion, a therapy that is currently entering the clinic to promote transplant tolerance.

## 1 Introduction

Solid organ transplantation (SOT) is the treatment of choice for patients with end stage organ failure. Despite improved short-term outcomes, the success of solid organ transplantation is limited by recipient immune responses (1–3). Therefore, there is a need to develop novel therapies to improve transplant outcomes.

In vascularized organ transplantation, evidence from animal studies suggests that coagulation proteases may play an important role in ischemia-reperfusion (IR) injury. Tissue factor (TF) expression was increased during experimental IR in rabbit heart (4), rat kidney (5) and liver (6) models and following cold storage of organs (7). Its importance in pathophysiology was supported by the observation that an anti-TF antibody or TF antisense oligonucleotides or a recombinant TF pathway inhibitor (TFPI), limited tissue necrosis, reduced inflammation and prevented leukocyte recruitment in some of the aforementioned models (4–6). Recently, a novel FXa/thrombin inhibitor was shown to reduce inflammation and significantly improve organ function in a porcine model of kidney transplantation after circulatory death (DCD) (8). In addition, the thrombin-formation inhibitor Melagatran has been shown to be beneficial in a porcine model of DCD kidney transplantation (9), by limiting leukocyte recruitment and reversing the vascular permeability seen in IR injury (10). Additionally, activation of protein C (PC) signaling *via* the thrombin receptor, protease receptor 1 (PAR-1), also protected against experimental lung and cardiac IR injury by inhibiting NLRP3 inflammasome activation (11, 12). Other studies have indicated that thrombin-dependent PAR-signaling is more important than fibrin deposition in IR injury (4). Moreover, activation of PAR-1 on graft dendritic cells (DCs) in IR has been linked to enhanced activation of alloreactive immune responses post-transplantation (13).

In addition, thrombosis is a key component of antibody-mediated rejection (AMR), and we have shown that inhibiting thrombin in a model of T-independent AMR is an effective way of prolonging organ survival (14). We have developed a novel class of membrane-localizing thrombin inhibitors called Thrombalexins (TLN) which contain hirulog, chemically modified to accept a lipid membrane-binding anchor. Different TLNs, when infused into donor organs prior to transplantation, can inhibit thrombosis mediated by AMR due to pre-existing donor-specific antibodies (DSA) in both rodent (15) and primate models (16). TLN-3, also known as PTL060, has been shown to stay localized to the endothelium within donor organs for up to 4 days (15, 16). Moreover, we have highlighted the non-thrombotic importance of thrombin, acting predominantly through PAR-1, in various models of acute and chronic vascular inflammation (17, 18), for the generation of local chemokine gradients, leading to recruitment of inflammatory leukocytes, and in the production of growth factors involved in hyperplastic vascular disease (19).

As described so far, while inflammatory responses involving thrombin are a threat to early transplant survival, particularly in the context of pre-existing DSA, transplant rejection in the absence of pre-existing DSA is dependent on activation of donor specific allogeneic T cells. Moreover, it is well established that the innate immune inflammatory responses amplify the adaptive alloimmune responses creating a bigger hurdle to the induction of tolerance (20–29).

CD4^+^CD25^+^Foxp3^+^ Tregs have a central role in the maintenance of immunological tolerance and the prevention of autoimmunity. Tregs and in particular donor-specific Tregs have been shown to inhibit transplant rejection (30–35). Signaling through innate receptors such as Toll like receptors (TLRs) (36–39) and anaphylatoxin receptors (C3aR/C5aR) (26, 29) has been shown to alter the function of Tregs. We have recently reported that PAR-4 expressed on murine Tregs can negatively regulate Treg function when activated by a high dose of thrombin or its agonist (23). All these observations suggest that the efficacy of Tregs may be compromised by tissue inflammation. Therefore, reducing innate immune responses should enhance the potential efficacy of Treg therapy as well as limiting the severity of the alloresponse.

In this study, we investigated whether combining the cytotopic thrombin inhibitor PTL060, with donor-specific Tregs was advantageous for the treatment of solid organ transplants. Our findings show that localized thrombin inhibition is effective in reducing early inflammation, in the absence of overt thrombosis, most likely by altering chemokine secretion and increasing recruitment of both anti-inflammatory macrophages and Tregs, thus inhibiting both cellular and humoral responses and, by increasing the efficacy of adoptively transferred Tregs, leading to improved heart transplant survival over that achieved by Tregs alone.

## 2 Materials and methods

### 2.1 Mice, reagents, and antibodies

C57BL/6 (B6) and BALB/c mice were purchased from Charles River. Procedures were carried out in accordance with all legal, ethical and institutional requirements and approved by the UK Home Office (PPL70/9066). The primary and secondary antibodies used in the current study are listed in **Supplementary Table 1**. PTL060 consists of membrane-localizing agent (mirystoyl tail) attached to the direct thrombin inhibitor hirulog (HLL) (15, 16, 40). PTL3146 is the membrane localizing agent, lacking the HLL moiety, used as a control for PTL060. RICS2 antibody (against HLL) was FITC conjugated for the detection of endothelium bound PTL060.

### 2.2 Induction of renal IR injury

Some mice received PTL060 (1 mg/kg) by i.v. injection one day prior to renal IR injury, as we previously described with some modification (41, 42). In brief, mice were anesthetized by isoflurane and a midline abdominal incision was made. The renal arteries and veins were bilaterally occluded with microaneurysm clamps for 30 minutes. After removal of the clamps, 1 ml of warm saline was put in the abdomen and the incision was sutured. The following day, some mice received 5 x 10^6^ polyclonally expanded CD45.1 Tregs i.v. At 48 hours after reperfusion mice were bled, culled and the kidneys harvested for histology, flow cytometry and Western blotting analysis. Serum samples were prepared from each animal to assess renal function using BUN concentrations measured with a Urea Assay Kit from Thermo Fisher (Epsom, United Kingdom).

### 2.3 Heart perfusion and transplantation

Donor hearts from BALB/c mice were harvested, under anesthesia following i.v. injection of 0.5 ml heparin *via* the inferior vena cava, residual heparin was flushed out with saline. Intracardiac perfusion, *via* the descending aorta, with 0.5 ml of 2 µM PTL060 or the control PTL3146, dissolved in Soltran perfusion solution (Baxter Healthcare Ltd, Newbury, UK), was performed for 15 min on ice. Following the perfusion, intra-abdominal heterotopic heart transplantation into B6 recipients was performed (34). Direct abdominal palpation of heterotopically transplanted hearts was used to assess heart graft viability and rejection was defined as the complete cessation of cardiac pulses. All the recipients received 200 µg of anti-CD8 antibody i.p. one day before and after transplantation, as previously described (34, 43–45). Some recipient mice received 5 × 10^6^ BALB/c specific Tregs by i.v. injection one day prior to transplantation. Graft survival between treatment groups was compared using the log-rank test.

### 2.4 Generation and expansion of donor specific CD4^+^CD25^+^ Treg lines

Bone marrow derived dendritic cells (BMDCs) were generated as previously described (34, 46). Briefly, bone marrow cells from BALB/c mice were treated with a mixture of anti-CD8 (YTS169), anti-CD4 (YTS191), anti-B220 (RA34.5), and anti–MHC class II (M5/114) antibody containing culture supernatants, before cells were washed and incubated with goat anti-rat IgG Dynal beads (ThermoFisher Scientific). Progenitor cells were isolated using a negative bead/magnet selection before being culturing with GM-CSF supplemented RPMI 1640 media. On days 2 and 4, of culture non-adherent cells were removed and fresh culture medium added. Cells were harvested after 6-day culture and irradiated with 3000 cG before use.

CD4^+^CD25^+^ T cells were enriched from the spleen and LNs of B6 mice using a Dynabead Flowcomp murine CD4^+^CD25^+^ Treg cell isolation kit (ThermoFisher Scientific) (34). The purity of CD4^+^CD25^+^ cells was over 95% (**Supplementary Figure 1A**). CD4^+^CD25^+^ Tregs (2 × 10^6^ cells/well) were stimulated weekly with irradiated immature BALB/c DCs (5 × 10^5^/well) in the presence of 10 U/ml recombinant human IL-2 (Proleukin-Novartis, Camberley, UK) in a 24-well plate. Donor specific suppressive capacity of the Tregs was assessed using ^3^H-thymidine incorporation, as previously published (47) with 1uCi of ^3^H-thymidine being added on day 2 of culture and incorporation measured 18-20 hours later (**Supplementary Figure 1B**).

### 2.5 Polyclonal expansion of CD4^+^CD25^+^ Treg lines

Enriched CD4^+^CD25^+^ T cells (2 × 10^5^ cells/well) from the spleen and LNs of CD45.1 mice, using the same protocol described above, were stimulated in a 24-well plate for 10 days with Dynabeads™ Mouse T-Activator CD3/CD28 (11456D, ThermoFisher) at a 1 to 3 (cell:bead) ratio in the presence of 2000 U/ml recombinant human IL-2 (Proleukin-Novartis, Camberley, UK). Beads were removed using a magnet 24 hours prior to adoptively transfer and bead free cells were maintained in the presence of IL-2.

### 2.6 Flow cytometric analysis

Kidney and cardiac cell suspensions were prepared using a previously described method (41, 42). In brief, the tissues were disaggregated and incubated in a solution including collagenase D (0.25 mg/ml), deoxyribonuclease (0.01 mg/ml), and FCS (10%) for 40 minutes at 37°C. The digested tissue was filtered through a 70 µm nylon cell strainer. The suspended cells were pre-incubated with anti-CD16/32 antibody (1 µg/ml), before being labelled using a LIVE/DEAD Fixable Near-IR Dead Cell Stain Kit (ThermoFisher Scientific). Cells were then stained with fluorescently conjugated CD45 (or CD45.1 and CD45.2), Gr-1, F4/80, CX3CR1, CD4, CD25 and FoxP3 antibodies at 4°C for 20 min as indicated in each experiment. In some cases, kidney or Treg cells were also stained with CCL17 or CCR4 as indicated, respectively. The cells were then fixed in 1% paraformaldehyde and acquired using a BD Fortessa Flow Cytometer (BD Biosciences, San Jose, CA) and analyzed using Flowjo software (Tree Star, OR, USA).

### 2.7 Histological analysis

To assess renal pathologic features, kidneys were fixed in a solution of 4% formalin, in PBS, for 24 hours before being embedded in paraffin. Sections (5 μm) were stained with hematoxylin and eosin and three fields per kidney were assessed using a magnification of ×200. Histologic score was performed as described previously (48) with some modification. Degrees of renal tissue injury was quantified as epithelial injury in the corticomedullary junction using a 7-point scale; 0, no injury; 1, 0%–15%; 2, 15%–30%; 3, 30%–45%, 4, 45%–60%; 5, 60%–75%; 6, >75% loss of proximal tubule brush border, cell blebbing or vacuolization, and cell necrosis.

Heart grafts were harvested 10 days after transplantation and embedded in paraffin, sectioned, and stained with hematoxylin and Eosin (H&E) or Verhoeff-van Gieson (EVG) for histological evaluation. To evaluate the degree of cellular infiltration, the number of cells in six independent fields, for each heart graft (six donor hearts for each group), were counted following H&E or EVG staining using a Zeiss AXIO Image Oberserver, with magnification x200 (Oberkochen, Germany). Alternatively, hearts were embedded in OCT and cryostat sections were processed and stained with FITC conjugated anti-CD68 antibody and either unconjugated rabbit anti-CCL17 antibody followed by AF647 conjugated goat anti-rabbit IgG, or with unconjugated rat anti-C4 antibody followed by AF568 conjugated goat anti-rat IgG, using the protocol established in our lab (23, 34). Anti-mouse C4 antibody binds to C4, C4b, and C4d, but because C4 is not cell bound, and C4b is short-lived, with an *in vitro* half-life measured in minutes (49), positive staining represents C4d deposition (50).

### 2.8 Western blot

Cell lysates were prepared from frozen kidney tissue using RIPA Lysis and Extraction buffer (ThermoFisher Scientific) supplemented with a protease inhibitor cocktail (Merck). Proteins were separated on pre-casted 4–12% SDS page gels (ThermoFisher Scientific) before being transferred to PVDF membranes. Blots were blocked in 5% skimmed milk before being incubated with primary antibodies specific for CCL17 or alpha-tubulin followed by secondary HRP-conjugated antibodies (Abcam), according to manufacturer’s recommendations. The membranes were developed with SuperSignal™ West Pico PLUS Chemiluminescent Substrate (ThermoFisher Scientific) and the results imaged by Bio-Rad Universal Hood II Gel Doc XR System. The protein levels were quantified by ImageJ (51).

### 2.9 Reverse transcription quantitative PCR (RT-qPCR)

Total RNA was extracted from heart and kidney samples using Trizol reagent (ThermoFisher Scientific). cDNA was synthesized with an RT kit (Promega, Southampton, UK). qPCR was performed using a DyNAmo HS SYBR Green qPCR kit (Bio-Rad, Watford, UK). Each sample was amplified in duplicates. The relative gene expressions of cytokine and chemokines were analyzed using the 2^-ΔΔ^ *^C^* ^T^ method and expressed as 2^-ΔΔ (Ct)^, where Ct is cycle threshold, ΔΔ (Ct) = testing samples, Δ (Ct) - control samples Δ (Ct); Δ (Ct) = testing gene (Ct) -18s (Ct) as described previously (21, 41). The basal control samples were taken from the normal BALB/c hearts or B6 kidneys. The information for primer sequences is listed in **Supplementary Table 2**.

### 2.10 Alloantibody detection

Anti-donor (BALB/c)-specific IgG levels present in serum samples taken from B6 heart transplant recipient mice were determined by flow cytometry using BALB/c splenocytes as target cells as described previously (52). Briefly, splenocytes were first stained with CD3-PE antibody (clone: 145-2C11) before incubating with serum samples (1:20 or 1:80 dilution) from recipient mice. Lastly cells were stained with FITC-conjugated rat anti-mouse IgG (Sigma-Aldrich Pool UK) and quantified by flow cytometry. Serum samples taken from non-transplanted B6 mice served as controls.

### 2.11 Statistics analysis

Statistical analysis was performed using GraphPad Prism. Data are expressed as mean ± SEM and statistical significance was determined using Two-way ANOVA Tukey’s multiple comparisons test as stated. In transplant experiments, statistical differences in the survival time were determined using a log-rank test. A difference was considered significant when p < 0.05.

## 3 Results

### 3.1 Inhibition of thrombin in combination with adoptively transferred Tregs during IRI protects renal function and reduces renal tissue injury by increasing recruitment of Tregs and reducing CX3CR1^+^ cell infiltration

**Figure 1:**
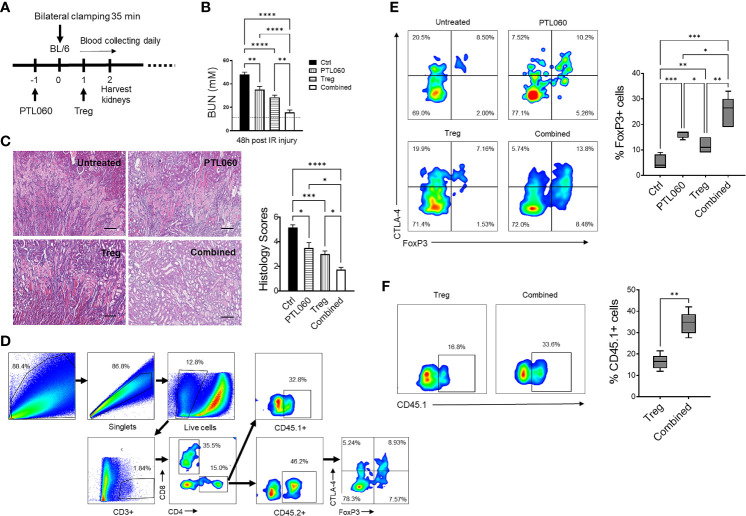
Administration of PTL060 or adoptively transferred polyclonal Tregs or the combination of the two protects mice from IRI with increased recruitment of Tregs into the ischemic kidneys. Renal IRI was induced in four groups of mice without or with treatments (n=6 per group, **A**). Serum samples and kidney tissues were collected at 48 hours after reperfusion. BUN levels were measured **(B)**. The dotted line indicates a normal BUN level. Light microscopic images of hematoxylin-eosin (H&E) show histopathologic features of injured kidneys. Representative light microscopic images of H&E of kidneys from the four groups at 48 hours after reperfusion **(C)**. Tissue injury was scored in cortical medullary junction areas at magnification of ×200 (n=6 per group three fields of each kidney, cumulative data in C). Single cell suspensions were prepared from the ischemic kidneys for flow cytometry with the gating strategy shown in **(D)**. The endogenous CD45.2^+^ **(E)** and adoptively transferred CD45.1^+^ **(F)** Tregs in the ischemic kidneys from the mice treated with PTL060 or injected with 5x10^6^ polyclonal Tregs or the combination of these two treatments were analyzed and compared to that in the Ctrl group in the cumulative graphs. Dead cells excluded using near IR live/dead cell staining kit. Data were analyzed by Two-way ANOVA Tukey’s multiple comparisons test. *p < 0.05, **p < 0.01, ***p < 0.005, ****p < 0.0001 in comparison between the four groups at 48 h post IRI or between the two Treg groups without or with PTL060.

Recent evidence has suggested that early infiltration of Tregs protects against ischemia reperfusion injury (IRI) and enhances repair process in IRI models of kidney (53, 54), heart (55, 56) and liver (57, 58). Using a murine renal IRI model, following the protocol described in **Figure 1A**, the impact of administration of PTL060 [which selectively tethers to endothelium after i.v. administration (59)], adoptively transferred polyclonal Tregs or the combination of both was evaluated. Renal function was assessed at 48 hours after reperfusion. All three treatments resulted in significantly lower levels of BUN compared with the IRI untreated group, indicating that renal function was protected (**Figure 1B**). The combination of both treatments gave the best protection. To confirm this renal tubular damage was assessed histologically in kidneys collected at 48 hours after reperfusion. Tubular injury consisting of tubule thinning, dilatation, loss of proximal brush border, and protein casts was noted in the IRI untreated group. However, all these were reduced in the treatment groups, with the greatest reduction in histological damage seen in the combined treatment group (**Figure 1C**).

**Figure 2:**
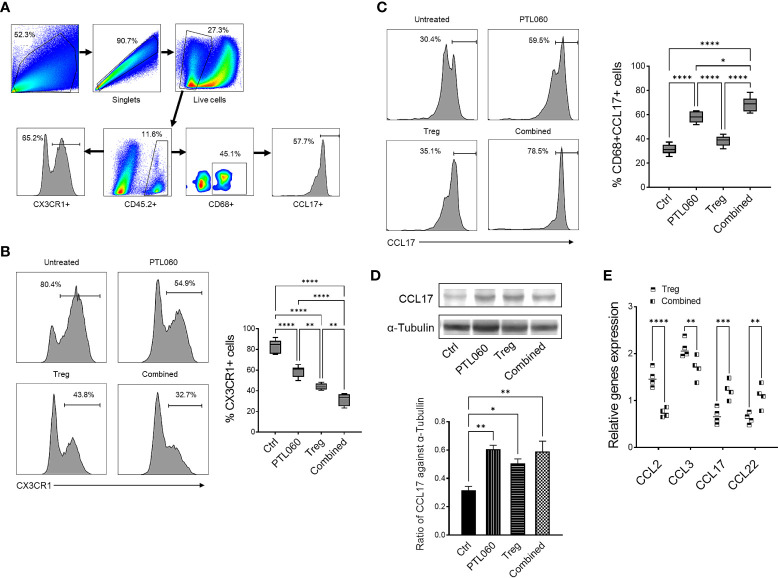
Administration of PTL060 or adoptively transferring of polyclonal Tregs or combined reduces the infiltration of CX3CR1^+^ cells and increases CD68^+^CCL17^+^ cells in the ischemic kidneys at 48 hours post IRI. Renal IR injury was induced in four groups of mice without or with treatments (n=6 per group). Single cell suspensions were prepared from the enzymatic digestion of the ischemic kidneys for flow cytometry with a gating strategy **(A)**. The CX3CR1^+^ **(B)** and CD68^+^CCL17^+^ **(C)** cells were analyzed and compared to that in the Ctrl group in the cumulative graphs (n=6). Dead cells excluded using near IR live/dead cell staining kit. Data were analyzed by Two-way ANOVA Tukey’s multiple comparisons test. *p < 0.05, **p < 0.01, ***p < 0.005, ****p < 0.0001 in comparison between the four groups at 48 h post IRI. The lysates prepared from the kidneys were used for Western blotting analysis. The protein levels of CCL17 in the ischemic kidneys were analyzed and quantified against alpha-Tubulin **(D)**. Graph shows mean ± SEM from four experiments (n=4). Data were analyzed by unpaired two-way t-test. *p < 0.05, **p < 0.01 in comparison between these four groups. RNA was extracted from the ischemic kidneys from each group and cDNA generated. qPCR was performed to assess gene profile of chemokines CCL2, CCL3, CCL17 and CCL22. Histogram graphs are shown for each chemokines **(E)**. Graphs represent mean ± SEM of the gene expression relative to the expression of 18S from four kidneys of each group (n=4). Data were analyzed by Two-way ANOVA Tukey’s multiple comparisons test. **p < 0.01, ***p < 0.005, ****p < 0.0001 in comparison between the four groups at 48 h post IRI.

Both adoptively transferred (CD45.1^+^) and endogenous Tregs (CD45.2^+^) could be detected 48h after reperfusion by flow cytometry (**Figures 1D–F**). Administration of PTL060 significantly enhanced the Treg recruitment whilst co-administration of PTL060 and Tregs resulted in significantly more CD45.2^+^ (**Figure 1E**) and CD45.1^+^ Tregs (**Figure 1F**) in the ischemic kidneys. Interestingly, the increased number of endogenous Tregs (CD45.2^+^) in the lymph tissue around renal pedicles and mesenteric lymph nodes in the treatment groups were observed in comparison to the untreated group, while the injected CD45.1^+^ Tregs were undetected in the same tissues (**Supplementary Figure 2A**). Meanwhile, compared to the untreated group the treatment groups had significantly lower infiltration of CX3CR1^+^ cells, which is one of major infiltrated cell type during the renal IR injury (60), with the lowest percentage of CX3CR1^+^ cells in the combination therapy (**Figures 2A, B**).

Consistent with what we have previously described (17, 18), there was significantly reduced CCL-2 (and CCL-3) expression in the kidneys of mice treated with PTL060 (**Figure 2E**).

However, PTL060 administration alone, promoted the recruitment of CD68^+^CCL17^+^ cells (M2 type of macrophage) into the ischemic kidneys with an increased percentages of these cells being observed. The highest percentages were observed when PTL060 was used in combination with Tregs (**Figure 2C**). Increased gene levels of the chemokines known to be involved in recruitment of Tregs such CCL17 and CCL-22 (**Figure 2E**) and increased protein level of CCL17 were detected in ischemic kidneys after treatment with PTL060 (**Figure 2D**). These results support the concept that PTL060 directly promotes Treg recruitment by skewing the expression of chemokines secreted during renal IRI.

### 3.2 Pre-transplant perfusion of PTL060 into donor hearts promoted allograft survival which was further enhanced by adoptive transfer of Tregs

**Figure 3:**
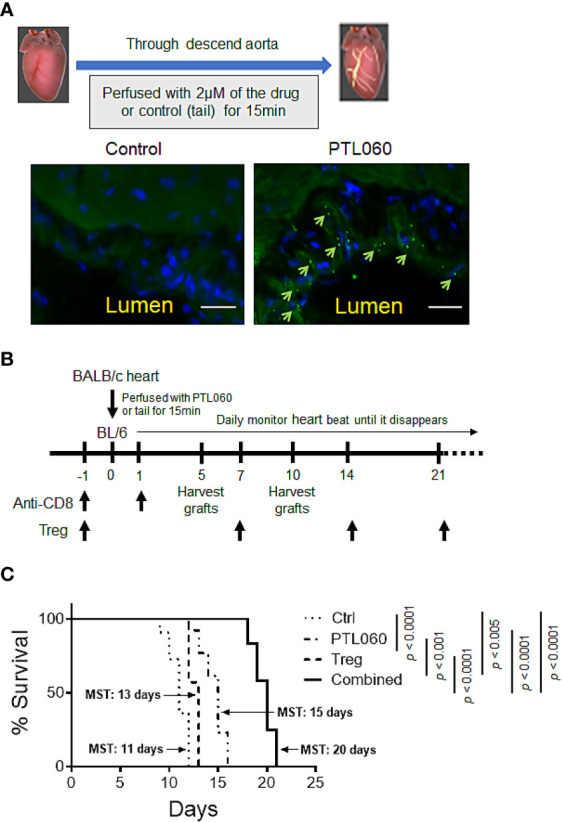
Perfusion of donor heart with thrombin inhibitor PTL060 or combined with adoptively transferring of Tregs prolongs allograft survival. Donor hearts from BALB/c mice were first perfused with 2 µM PTL060 or treated with the tail as control (Ctrl), for 15 min on ice before transplantation. OCT sections of donor heart grafts were stained with FITC-conjugated HLL peptide (green) and DAPI (blue) and samples visualized using a fluorescence microscope (scale bar: 25 µm). Representative images of PTL060 treated hearts compared to the Ctrl are shown, where PTL060 binding is highlighted using arrows **(A)**. Schematic representation of the timeline of experimental procedures undertaken for allogeneic BALB/c heart transplantation **(B)**. BALB/c hearts perfused with PTL060 or with the Ctrl were transplanted into B6 recipients (n=11 & n=14, respectively). Some Ctrl and PTL060 mice received 5 x 10^5^ BALB/c specific Tregs by i.v. injection every seven days (Treg group, n=10 & Combined group, n=12). Transplanted hearts were monitored daily and rejection was defined as the ceasing of the donor heart beating **(C)**. Statistical analysis of heart graft survival was performed using a Log-rank test. ***p < 0.005, ****p < 0.0001 in comparison between the four groups.

To assess the impact of PTL060 on allograft outcome, we switched to a murine heterotopic heart transplant model as the timing of rejection is more easily defined by monitoring the heart beats, at which point the animal is usually healthy. Additionally, a transplant model allows treatment of the donor endothelium by perfusing the organ with PTL060 prior to transplantation, rather than i.v. injection. First, we evaluated whether PTL060 effectively bound to heart endothelium as observed in other organs such as kidneys (15, 16). OCT sections of donor hearts perfused with PTL060 or control (Ctrl) were stained with a FITC-conjugated RICS2 antibody. PTL060 was detected on the luminal surface of blood vessels in the heart (**Figure 3A**).

Next, to test the impact of thrombin inhibition on allograft rejection and the potential benefit of combining with Treg administration, PTL060 treated BALB/c hearts were transplanted into B6 mice (**Figure 3B**). All the recipient mice received anti-CD8 antibody the day before and day after transplantation to eliminate the contribution of CD8^+^ T cells with direct allospecificity, as previously published (34). This allows us to assess the actions of PTL060 on CD4^+^ T mediated rejection. Perfusion of donor hearts with PTL060 significantly prolonged allograft survival (Mean Survival Time (MST) = 15 days) compared to the control group (MST = 11 days, **Figure 3C**). Having shown previously that donor-specific Tregs can increase heart transplant survival (34), we explored whether pre-treatment with PTL060 could further enhance their efficacy. To test this, BALB/c specific Tregs were adoptively transferred into B6 recipients the day before receiving donor hearts treated with PT060 or Ctrl (**Figure 3C**). As expected Treg treatment extended heart transplant survival from 11 to 13 days whereas combined with PTL060 treatment heart survival was extended to 20 days. Compared to the control, BALB/c hearts treated with PTL060 alone or in combination with Tregs appeared visually normal, both in size and shape, at day 5 or 10 after transplantation (**Supplementary Figure 3**).

### 3.3 Inhibiting thrombin in the presence of Tregs reduced cell infiltration and tissue inflammation

**Figure 4:**
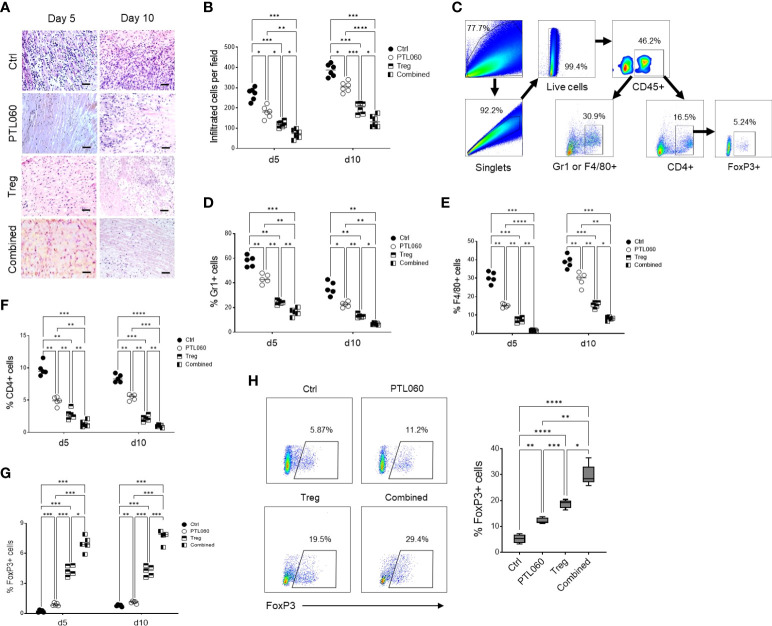
Combined treatment has further reduced cell infiltration and inflammation. Donor hearts treated with PTL060 or untreated (Ctrl) were transplanted into B6 recipients. Some recipient mice were injected with 2.5x10^6^ Tregs. Allografts were harvested at day 5 or 10 after transplantation. The grafts were processed in formalin and stained with H&E. A representative histology was shown from donor grafts treated with PTL060 or Ctrl with or without receiving Tregs **(A)**. Scale bars: 50 µm. Cumulative graph of infiltrated cells with magnification x200. Graph represents mean±SEM from six donor hearts for each group **(B)**. Donor grafts were also enzymatically digested for flowcytometry analysis with a gating strategy **(C)**, and the cells stained with conjugated antibodies CD45.2, Gr-1, F4/80, CD4, CD25 and FoxP3 followed by flow cytometry analysis and cumulative graphs of each subpopulation are shown in **(D–G)**, respectively. The CD4+FoxP3+ cells in the peripheral LNs from the recipients receiving the heart grafts either treated with PTL060 or injected with BALB/c specific Tregs or the combination of these two treatments at day 5 post transplantation were analyzed and compared to that in the Ctrl group in the cumulative graph **(H)**. Dead cells excluded using near IR live/dead cell staining kit. Live CD45+ cells were gated for further analysis. Graph represents mean±SEM from four donor hearts each group. Data were analyzed by Two-way ANOVA Tukey’s multiple comparisons test. *p < 0.05, **p < 0.01, ***p < 0.005, ****p < 0.0001 in comparison between the four groups at day 5 or 10.

To assess the impact of the combined treatment on tissue damage and understand the mechanisms behind the extended transplant survival, donor hearts were examined histologically at day 5 and 10 after transplantation. Compared to the untreated hearts (Ctrl), improved tissue structure and reduced cellular infiltration was observed in PTL060-perfused hearts as well as following adoptive transfer of Tregs alone (**Figures 4A, B**). Flow cytometric analysis of cells, following the gating strategy (**Figure 4C**), present in the donor hearts at day 5 and 10 after transplantation showed that when the hearts were transplanted after PTL060 perfusion a significantly reduced percentages of neutrophils and inflammatory monocytes (Gr-1^+^), macrophages (F4/80^+^) and CD4^+^ cells. These reductions were even more evident when the hearts obtained from recipient mice injected with Tregs, compared to the untreated hearts (**Figures 4D–F** respectively). In contrast, significant increases in FoxP3^+^ cells of CD4^+^ populations were observed following PTL060 or Treg or the combination treatment compared to the mice receiving the untreated hearts (**Figure 4G**). This finding was confirmed by immunofluorescence staining of donor hearts section at day 5 and 10 after transplantation (**Supplementary Figures 4A, B**).

As expected, the combined treatment further reduced cellular infiltration. In fact, the percentage of Gr1^+^ and F4/80^+^ cells as well as CD4^+^ T cells into donor hearts was significantly reduced compared to the hearts perfused with PTL060 or hearts from mice injected with Tregs alone (**Figures 4D–F** respectively). By contrast, the numbers of FoxP3^+^ cells within the hearts were the highest among all the hearts analyzed from the other groups of mice (**Figure 4G** and **Supplementary Figures 4A, B**). Moreover, peripheral LNs were also analyzed at day 5 post transplantation. Both PTL060 treatment and adoptive antigen-specific Treg treatment alone led to an increase in CD4^+^Foxp3^+^ T cells (**Figure 4H**). Most importantly, combining PTL060 treatment with Treg infusion promoted further Tregs migrating into the LNs, which could decrease allo-immune responses.

Finally, when the hearts were analyzed at the time of rejection, in each group of mice, a reduced number of Tregs was observed compared to day 5 and day 10, while the CD4^+^Foxp3^-^ T cells were increased (**Supplementary Figures 4A, B**). The results so far are suggesting that PTL060 treatment of the hearts before transplantation and Tregs alone led to a decrease in Gr1^+^, F4/80^+^ and CD4^+^ T cells while CD4^+^Foxp3^+^ T cell were increased, although transiently. Most importantly, combining PTL060 pretreatment of the hearts with Treg infusion reduced further tissue injury and leukocyte infiltration whilst favoring Treg accumulation in the heart leading to increased heart transplant survival.

### 3.4 PTL060 treatment alone or in combination with Tregs alter pro-inflammatory mediators and chemokine expression in donor hearts

**Figure 5:**
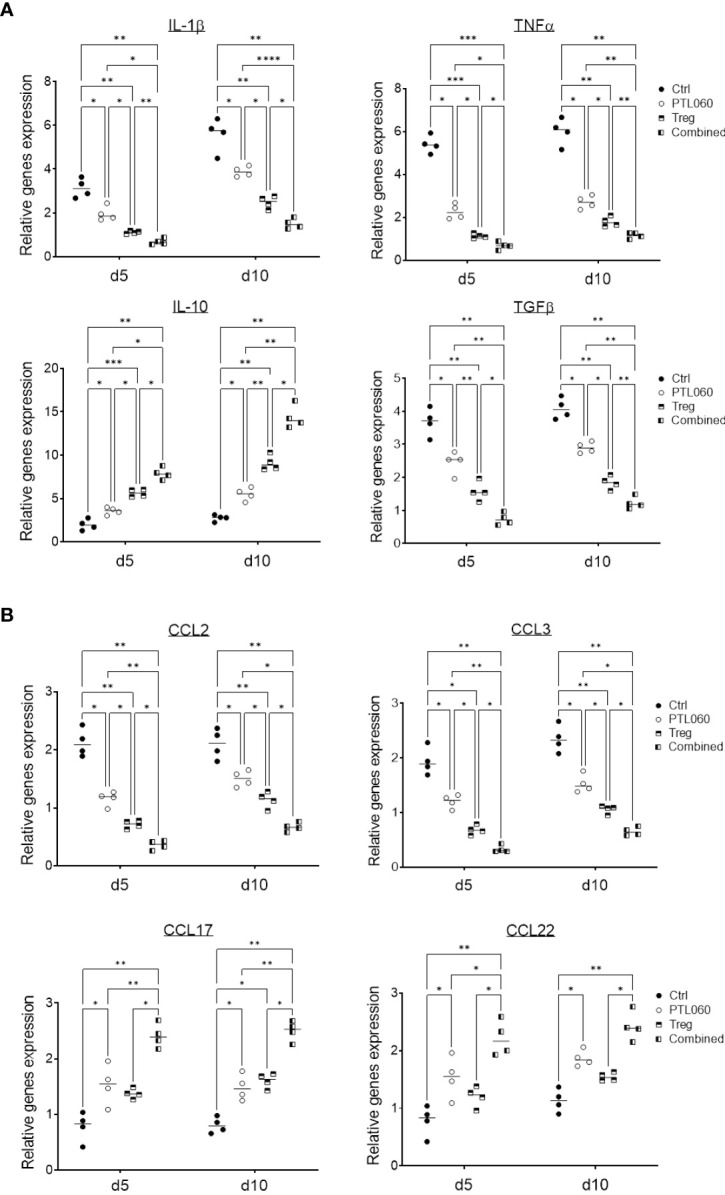
Gene profile of cytokine and chemokine production in PTL060 treated heart allografts without or with Treg infusion. Untreated (Ctrl) or PTL060 treated donor hearts without or with adoptively transferred Tregs in recipient mice, were harvested at day 5 or 10 after transplantation. RNA was extracted from the allografts from each groups and cDNA generated. qPCR was performed to assess gene profile of cytokines (IL-1β, TNFα, IL-10 and TGFβ) and chemokines (CCL-2, CCL-3, CCL-17 and CCL-22). Histogram graphs are shown for each cytokine **(A)** and chemokines **(B)**. Graphs represent mean ± SEM of the gene expression relative to the expression of 18S from four donor hearts from each group. Data were analyzed by Two-way ANOVA Tukey’s multiple comparisons test. *p < 0.05, **p < 0.01, ***p < 0.005, ****p < 0.0001 in comparison between the four groups at day 5 or 10.

The production of inflammatory mediators in the donor hearts with the single or combined treatment was assessed by RT-qPCR at day 5 and 10 post-transplantation. Compared to controls, the donor tissues from hearts perfused with PTL060 before transplantation or from mice receiving Treg infusion exhibited significantly lower levels of the pro-inflammatory cytokines IL-1β, TNFα and TGFβ (**Figure 5A**) and chemokines CCL-2 (MCP-1) and CCL-3 (MIP1α, **Figure 5B**). Conversely, there was significantly higher expression of the cytokine IL-10 (**Figure 5A**) and chemokines CCL-17 and CCL-22 in PTL060 and Tregs-treated hearts compared to the control group (**Figure 5B**). These changes were further enhanced when Tregs and PTL060 were combined (**Figures 5A, B**).

These findings indicate that inhibition of thrombin in the vasculature of donor hearts before transplantation selectively reduced the expression of chemokines involved in effector T cell recruitment and promoted the recruitment of CCR4 bearing cells including Tregs (**Supplementary Figure 5**). Indirectly, these findings support the concept that, even in the absence of overt thrombosis, thrombin-mediated inflammation at the vascular interface is operating to promote inflammatory cells but suppressing Treg recruitment.

### 3.5 Increased number of CD68^+^CCL17^+^ macrophage subset in the heart allografts after the perfusion of PTL060 without or with the infusion of Tregs

**Figure 6:**
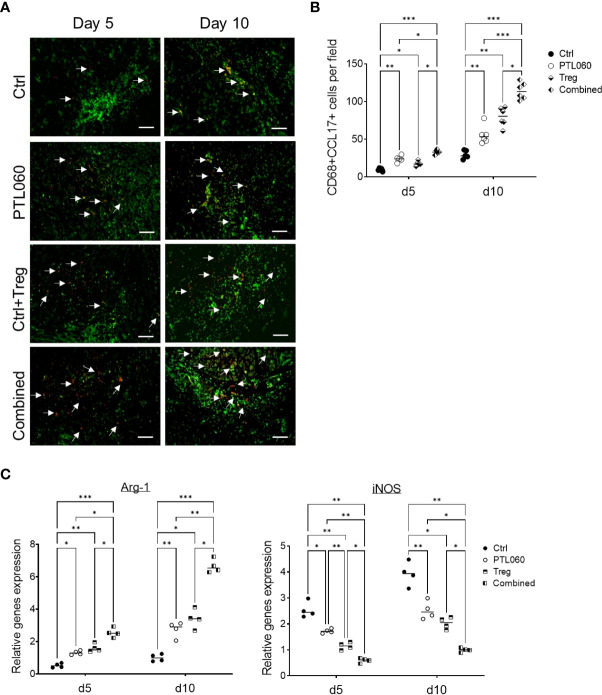
Characterization of macrophage subsets in heart grafts. Some recipients, transplanted with donor hearts treated with PTL060 or Ctrl, were also injected with Tregs. Heart grafts were harvested at day 5 or 10 after transplantation. Representative photomicrographs of OCT sections of the donor hearts stained with CD68 (Green), CCL-17 (red) and DAPI for nucleus (blue) from each treatment group **(A)**. Cumulative graph of the number of CD68+CCL-17+ cells per field with magnification x400 in the donor heart from each group **(B)**. Graph represents mean±SEM from four donor hearts for day 5 (n=4) and from six donor hearts for day 10 (n=6) from each group. Scale bars: 25 μm. RNA was also extracted from the donor hearts from each group and cDNA generated. qPCR was performed to assess gene profile of arginase I (Arg-1) and iNOS shown in cumulative graph **(C)**. Graph represents mean±SEM of the gene expression relative to the expression of 18S from four donor hearts each group. Data were analyzed by Two-way ANOVA Tukey’s multiple comparisons test. *p < 0.05, **p < 0.01, ***p < 0.005 in comparison between the four groups at day 5 or 10.

Recent evidence has suggested that macrophage derived chemokines CCL-17 (and CCL-22) play a central role in recruiting Tregs to inflammatory sites (61–65). Given that these chemokines were increased in PTL060 treated hearts, further analysis was performed to examine whether the macrophages in the PTL060 perfused grafts produced CCL-17 (66–68). CD68 has been previously used to examine macrophage infiltrates (69–71). Cells expressing both CD68 and CCL-17 were analyzed using immunofluorescence staining, on days 5 and 10 after transplantation and compared between the four groups of mice. There were significantly more CD68^+^CCL-17^+^ cells in the grafts from mice transplanted with either PTL060-perfused hearts of mice injected with Tregs compared to the control group at day 10 after transplantation, but the highest number of this subset was found after combined therapy (**Figures 6A, B**). To complement these observations, the expression of arginase (Arg-1) and inducible nitric oxide synthase (iNOS), previously used to characterize macrophage polarization (72–74), were analyzed by RT-qPCRs at day 5 and 10 after transplantation. Hearts pretreated with PTL060 or hearts from recipient mice injected with Tregs alone showed significantly increased expression of Arg-1, associated with M2 macrophages and decreased levels of iNOS, associated with M1 macrophages, compared to the control group (**Figure 6C**). As expected, combined treatment further enhanced these changes. Together, these results suggest that both PTL060 and donor specific Tregs promote recruitment and differentiation of M2 type of macrophages which secrete chemokines to further enhance Treg recruitment.

### 3.6 Combined treatment delayed graft-injurious humoral responses in the recipients following transplantation

**Figure 7:**
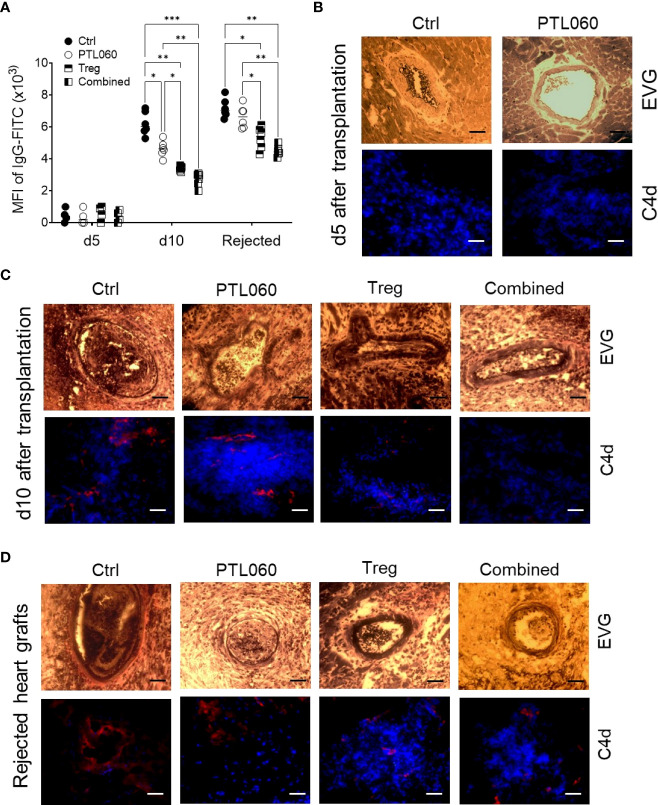
Development and characterization of antibody-mediated heart allograft rejection. Allo-antibodies (IgG) were measured in the serum of the recipients using a flow cytometry protocol at day 5 and 10 after transplantation or when the heart grafts were rejected **(A)**. Histological analysis of humoral rejection in the recipients was also performed. Representative photomicrographs of the allografts with EVG staining (paraffin sections) or immunofluorescence staining for C4d (red) and nucleus with DAPI (blue, OCT section) at day **(B)** or day 10 **(C)** or at the day the allografts rejected **(D)**. Scale bars: 25 μm. Graphs represents MFI±SEM from four to six recipients from each group. Data were analyzed by Two-way ANOVA Tukey’s multiple comparisons test. *p < 0.05, **p < 0.01, ***p < 0.005, in comparison between the four groups at day 5 or 10 or at the day the allografts rejected.

We have previously shown that tethered thrombin inhibitors like PTL060, by inhibiting intravascular thrombosis could delay acute antibody-mediated rejection in a rat hyperacute rejection model (15). Although rejection in the murine heart transplant model applied in the current study has been shown to involve a dominant cellular alloresponse, the humoral response was also assessed. The serum samples were collected at day 5 and 10 after transplantation, or at the time of graft rejection, and the levels of allo-IgG assessed using a flow cytometry protocol (50, 75). Very little allo-IgG antibodies were detected in the sera at day 5 (**Figure 7A**). However, the level of allo-IgG in the sera of recipient mice receiving one of the treatments were significantly lower than that of the untreated control group at day 10 after transplantation, with the lowest IgG levels detected in the mice receiving combined PTL060 and Treg treatment. Despite this, by rejection, all four groups had significant levels of circulating DSA, however the lowest levels of allo-IgG were observed in the mice receiving combined therapy.

Histological assessment of the grafts from the four groups of mice at day 5 and 10 post-transplant showed that vasculopathic process was evident in control hearts, with arterial intimal thickening, diffuse myocyte loss and fibrosis, together with C4d deposition at day 10, but not at day 5 after transplantation, suggesting a humoral response had occurred after 5 days post transplantation and contributed to graft rejection in this model. These pathological changes were significantly mitigated with the single or combined treatments (**Figures 7B, C**), particularly when Tregs were used. Interestingly, the levels of allo-IgG in the recipients after the grafts were rejected were also significantly lower, with reduced C4d deposition within the allografts, following a single or combined treatment (**Figures 7A, D**), suggesting that the single treatment, particularly Treg infusion, and the combination of thrombin inhibition and Treg therapy, were able to delay the humoral response. Nevertheless, hearts from all groups showed features of antibody mediated rejection on the day of rejection. These data suggest that adoptively transferred donor-specific Tregs, particularly when combined with perfusion of the organ with PTL060 pre-transplant, can inhibit the resulting cellular inflammation within the graft but that single treatment with PTL060 only transiently delay the development of the humoral response, which ultimately contributed to graft rejection.

## 4 Discussion

In this study, we have shown that PTL060 administration prior to renal IR injury and pre-transplant perfusion of BALB/c hearts with PTL060 significantly protected against acute renal injury and prolonged graft survival in B6 recipient mice. This was associated with a reduced infiltration of pro-inflammatory cells, including neutrophils, macrophages and T cells leading to reduced tissue inflammation. The significant reduction in expression of CCL2 and CCL3 suggested that PTL060 inhibited the local chemokine gradients involved in monocyte and CD4^+^ T cell recruitment, as previously demonstrated (17, 18). Surprisingly, and for the first time, we have shown that PTL060-treated kidneys and hearts contained increased numbers of Tregs, in association with increased expression of CCL-17 and -22, the chemokines involved in Treg recruitment. These data support the hypothesis that in control animals, thrombin inhibits the generation of these chemokines and thus inhibits Treg recruitment. The fact that PTL060 could enhance the Treg recruitment as early as 48h after IR injury suggests that these changes in recruitment are also initiated by the IRI associated with transplantation.

In addition, thrombin inhibition by PTL060 skewed the polarization of macrophages in the graft toward an M2 phenotype; some of these cells expressed CCL-17 which may have contributed to enhancing Treg recruitment. We acknowledge that our data would have been significantly strengthened by *in vitro* work looking at how thrombin, and conversely inhibition of thrombin, influenced myeloid cell phenotype. However, these data are entirely consistent with those we have previously described in a non-transplant model, in which weekly administration of PTL060 in ApoE^-/-^ mice caused reduced monocyte recruitment into atheromatous plaques, where they developed an M2-like phenotype associated with regression of atheroma (59). Moreover, in more recent work, we did an extensive *in vitro* investigation using BM-derived macrophages to show how thrombin primed macrophages become exquisitely sensitive to M1 polarizing cues, *via* ABCA1-mediated changes in cell membrane cholesterol-rich microdomain expression (76, 77).

We have previously shown, using the same mouse heart transplant model, that adoptive transfer of Tregs with direct allospecificity, for H-2^d^ alloantigens, delays rejection but did not promote long-term graft survival (34). In this paper, we show that adoptive transfer of Tregs with direct allospecificity appears to promote similar changes as PTL060 treatment. Importantly, the combination of PTL060 with Tregs had an additive impact on chemokine production, leukocyte recruitment and phenotype and cardiac survival.

Cardiac inflammation, triggered by activation of innate immune cells as well as non-immune cells (e.g. endothelial cells) as defense mechanism against tissue stress and injury, contributes to cardiac dysfunction and fibrosis after transplantation. Identification of key mediators of inflammatory responses driving tissue injury and fibrotic process after transplantation may lead to improvement of heart graft function through specific interventions. Although there is compelling evidence on the pathogenic roles of thrombin, a key serine protease in hemostasis, in acute cardiac IR injury (78, 79), chronic vasculopathy (18), graft dysfunction and allograft rejection (14), the impact of the thrombin activation on the donor graft, leading to allograft rejection and progression of cardiac fibrosis has been unclear. The present study provides evidence, for the first time, that thrombin mediated signals, most likely related to the IRI associated with transplantation, promote the development of cardiac inflammation in the absence of overt thrombosis.

PAR activation by thrombin and/or other serine proteases could regulate adaptive immune responses by targeting immune cells such as effector T cells (23, 80–82) as well as antigen presentation cells such as DCs (80, 83). We have previously shown that T cell priming is not influenced by thrombin inhibition in a model of alloimmune-mediated arteriosclerosis (84) and another model of contact hypersensitivity after oxazolone exposure (76). However, in this work, we have not directly addressed the impact of thrombin, nor thrombin inhibition on the interactions between alloreactive CD4^+^ T cells and either graft-derived or recipient-derived APC and this is a potential weakness which we acknowledge. Instead, we have associated the major effects of PTL060 in the heart as being related to the increased number of Tregs seen. Our findings of increased intragraft expression of chemokines CCL-17 and CCL-22, increased CD68^+^CCL-17^+^ M2 macrophage subset and increased FoxP3^+^ Tregs in the recipients with the single or combined treatment are consistent with PAR activation preventing the intragraft recruitment of CD4^+^FoxP3^+^ cells and thus we propose that the major impact of PTL060 is a direct skewing of chemokines away from those involved in recruitment of inflammatory myeloid cells (CCL2) towards those responsible for recruitment of Tregs. Our data is consistent with the observation of Tregs reducing IR injury of kidney (53, 54), heart (55, 56) and liver (57, 58). We have recently published that Tregs that lack PAR4 expression have an increased function, suggesting that inflammation mediated by thrombin can negatively regulate Tregs (23). Thus, it is possible that PTL060 could also be directly influencing the potency of the recruited Tregs by preventing signaling through PAR-4. We have not dissected the relative contribution of these two mechanisms, which we acknowledge is another limitation of our presented findings, nevertheless, our data suggest that the combination of these two effects on thrombin inhibition combine to have significant impact on heart transplant survival.

The failure to observe indefinite allograft survival with the model applied in the current study is likely due to several factors. Firstly, our previous work suggests that Tregs with direct allospecificity are incapable of promoting long-term allograft survival following adoptive transfer in this model as the indirect alloresponse was not suppressed (34). Since this pathway is the main driver of humoral immunity, and we saw high levels of circulating DSA in all groups beyond day 5 (albeit delayed in our treatment groups), we conclude that AMR contributed to graft loss. Secondly, although we have previously shown that thrombin inhibition inside the graft is an effective way to inhibit AMR (15), we know that a single dose of PTL060 applied to the graft prior to transplantation protects that endothelium for up to 4 days, but no longer, which is before the humoral response was detectable in all our groups. Our prediction is that repeated doses of PTL060, given *in vivo* for instance weekly, would significantly protect the graft from the developing humoral response, but this has not yet been tested. Thirdly, in this study and different from our previously published work using the same model, we adoptively transfer Tregs in the absence of rapamycin, as additional therapy (34).

Our findings demonstrate a pathogenic role for thrombin after vascularized transplantation in a model where rejection is dependent on activation of CD4^+^ T cells and does not involve a thrombotic phenotype. The mechanism involves inhibition of pro-inflammatory cell recruitment and preferential recruitment of Tregs, *via* an effect on chemokine gradient expression and on the phenotype of recruited myeloid cells. Inhibition of thrombin inside the graft, using a novel reagent PTL060, significantly enhances the impact of adoptively transferred Tregs on graft survival. Although the humoral response is delayed, it is not inhibited in this model so rejection is not prevented. This interpretation of the data is supported by parallel studies in a model of delayed type hypersensitivity, that we have just reported (76). In this model, thrombin plays a critical role by priming macrophages to be exquisitely sensitive to polarization by IFNγ. Altogether, this is a novel therapeutic approach, which can be further developed to treat excessive immune activation in autoimmune disorders, chronic inflammatory conditions, and allograft rejection.

## Data availability statement

The original contributions presented in the study are included in the article/**Supplementary Material**, further inquiries can be directed to the corresponding author/s.

## Ethics statement

The animal study was reviewed and approved by AWERB, King’s College London.

## Author contributions

QP conception and design, collection and assembly of data, data analysis and interpretation, manuscript writing. AN performing mouse heart transplantation. KR technical support. LS intellectual input, critical revision of the article. RS designing thrombin inhibitor PTL060, intellectual input, critical revision of the article. RL and AD intellectual input, critical revision of the article. GL conception and design, critical revision of the article for important intellectual contents. All authors contributed to the article and approved the submitted version.
