# Supplementary material for: Inhibition of thrombin on endothelium enhances recruitment of regulatory T cells during IRI and when combined with adoptive Treg transfer, significantly protects against acute tissue injury and prolongs allograft survival
Source: Front Immunol. 2023 Jan 30;13:980462. doi: 10.3389/fimmu.2022.980462 (PMC9924086; doi:10.3389/fimmu.2022.980462)
Supplement: Supplementary file 1 [file DataSheet_1.pdf]

Supplementary Table 1. Information of anti-mouse antibodies used in the current study.

Antibodies	Clones	Conjugates	Manufacturers	Catalogue numbers
CD45	30-F11	FITC	ThermoFisher	11-0451-82
Cd45.1	A20	BV421	BioLegend	110731
CD45.2	104	PerCP	BioLegend	109826
CD3ε	145-2C11	FITC	BioLegend	100306
CD4	GK1.5	PE-Cy7	BioLegend	100422
CD25	PC61.5	BV605	BioLegend	1020351
CD8	53-6.7	BV605	BioLegend	100743
FoxP3	FJK-16s	PE	ThermoFisher	12-5773-82
CD152 (CTLA-4)	UC10-4B9	APC	ThermoFisher	17-1522-82
CD68	FA-11	FITC	BioLegend	137005
F4/80	BM8	PE	BioLegend	157304
Gr-1	RB6-8C5	PE	ThermoFisher	12-5931-82
CCR4	2G12	PE	BioLegend	1312031
Rat anti-C4	16D2	Primary	abcam	ab11863
CCL17	polyclonal	Primary	Biorbyt	Orb412139
Rabbit anti-α-Tubulin	polyclonal	Primary	Sigma-Aldrich	SAB4500087
Anti-rabbit IgG	AF647		abcam	ab150075
Anti-rat IgG	AF568		abcam	ab175475
Anti-rabbit IgG	HRP		abcam	Ab6721

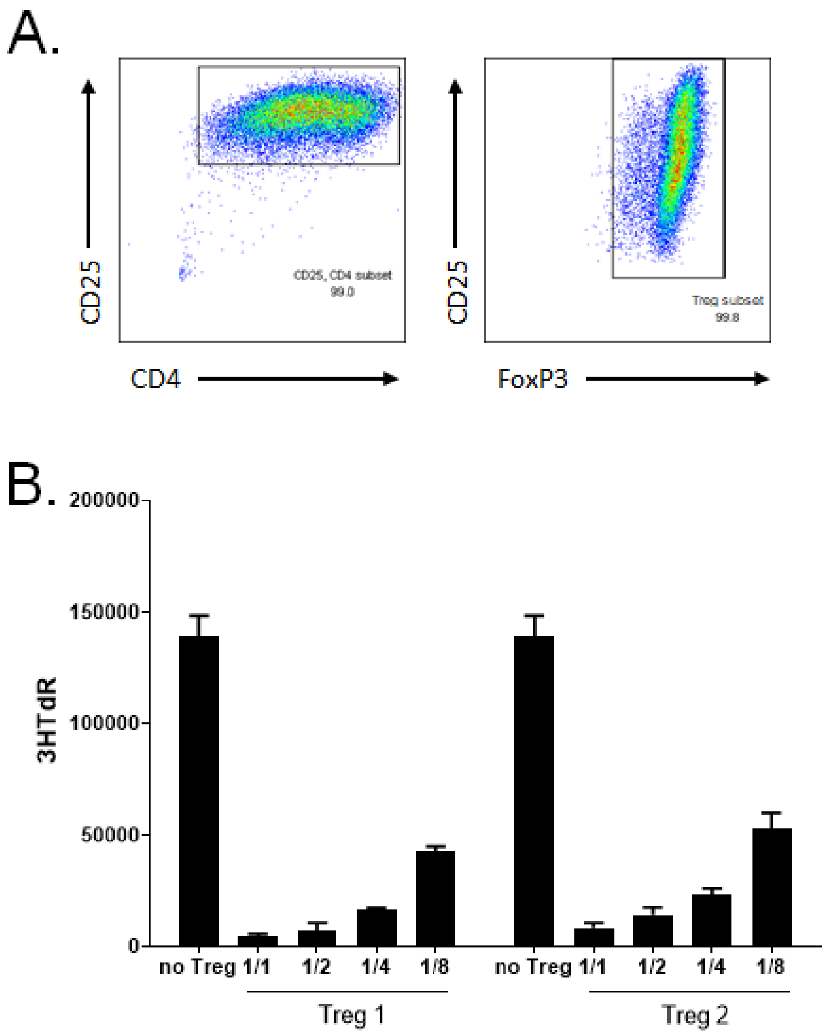

Suppl Figure 1. Purity and suppressive capacity of expanded donor specific Treg. The purity of the enriched CD4$^+$CD25$^+$ T cells from the spleen and LNs of B6 mice was assessed (A). The BALB/c specific Tregs were generated and expanded weekly with irradiated immature bone marrow-derived BALB/c DCs in the presence of 10 U/ml recombinant human IL-2 for six weeks. The specific suppressive capacity in different Treg:Tresp ratios was assessed with ^{3}H-thymidine incorporation after 3 days of culture. The representatives of ^{3}H-thymidine uptake from two expanded Tregs were shown (B).

Supplementary Table 2. PCR primer sequences and product sizes PCR primer sequences and product sizes

Primer	Oligonucleotide Sequence (5’ → 3’)	Product Size (bp)	Gene bank code
18S-1<br>18S-2	ATC CCT GAG AAG TTC CAG CA<br>CCT CTT GGT GAG GTC GAT GT	153	NM_011296.1
IL-1β-1<br>IL-1β-2	GCTCTCCACCTCAATGGACA<br>TTGGGATCCACACTCTCCAG	182	ENSMUSG00000027398
TNFα-1<br>TNFα-2	TGAGCACAGAAAGCATGATCC<br>GCCATTTGGGAACTTCTCATC	200	ENSMUSG00000024401
IL-10-1<br>IL-10-2	CCAAGCCTTATCGGAAATGA<br>TTTTCACAGGGGAGAAATCG	162	ENSMUST00000016673
TGFβ-1<br>TGFβ-2	AAT ACG TCA GAC ATT CGG GAA<br>CCG GGT TGT GTT GGT TGT AGA G	640	NM_011577
MCP-1-1*<br>MCP-1-2	GGCTCAGCCAGATGCAGTTA<br>ATTTGGTTCCGATCCAGGTT	219	ENSMUSG00000035385
MIP-1α-1**<br>MIP-1α-2	CACTGCCCTTGCTGTTCTTC<br>GGCATTCAGTTCCAGGTCAG	262	ENSMUSG00000000982
CCL17-1<br>CCL17-2	GCTCTGCTTCTGGGGACTTT<br>ACCAGCTCACCAACTTCCTG	121	MGI:1329039
CCL22-1<br>CCL22-2	CAAGCCTGGCGTTGTTTTGA<br>TAGAGGGACCAGAGCCTCAC	250	ENSMUSG00000031779
Arg1-1<br>Arg2-2	TCAACACTCCCCTGACAACC<br>TCTACGTCTCGCAAGCCAAT	163	NM_007482.3
iNOS-1<br>iNOS-2	GGTTGTCTGCATGGACCAGT<br>TTCAGAGTCTGCCCATTGCT	114	NM_010927.4

*MCP-1 = CCL2; **MIP1α = CCL3.

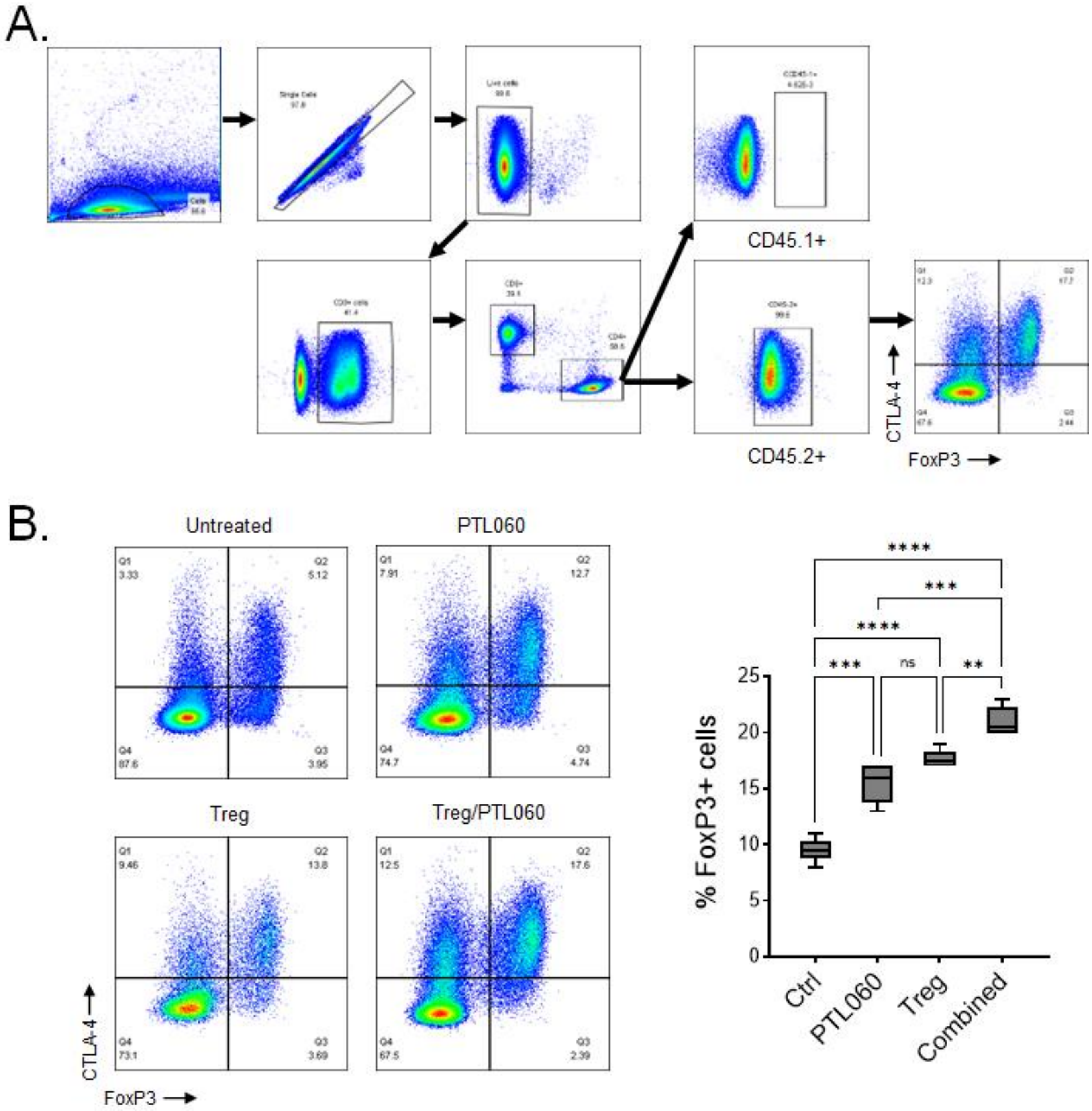

Suppl. Fig 2. Combined treatment has further increased endogenous Tregs into the peripheral lymph tissues. Endogenous Tregs in the digested ischemic kidney cells were assessed by flow cytometry with the gating strategy presented in (A). The endogenous Tregs in the peripheral lymph tissues from the mice treated with PTL060 or injected with $5x10^5$ polyclonally expanded Tregs or the combination of these two treatments were analyzed and compared to that in the Ctrl group in the cumulative graph (B). Dead cells excluded using near IR live/dead cell staining kit. Data were analyzed by Two-way ANOVA Tukey's multiple comparisons test. ** $p < 0.01$, *** $p < 0.005$, **** $p < 0.0001$ in comparison between the four groups at 48 h post IRI.

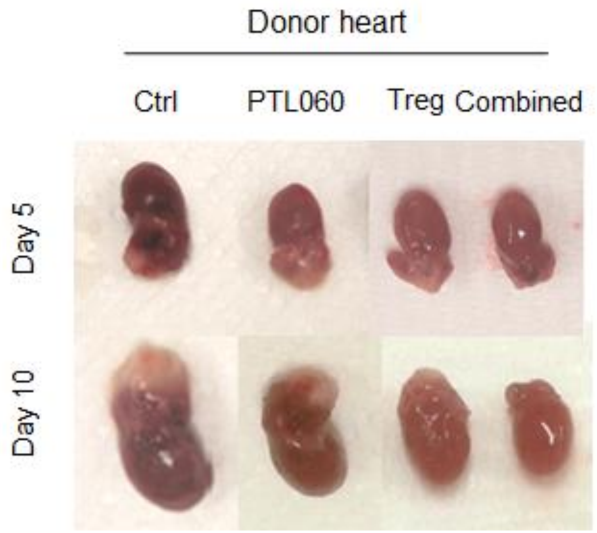

Suppl. Fig 3. Donor hearts with the single or combined treatment. The representative images of donor hearts with the single (PTL060 or Treg alone) or combined treatment and the control were harvested at day 5 and 10 after transplantation.

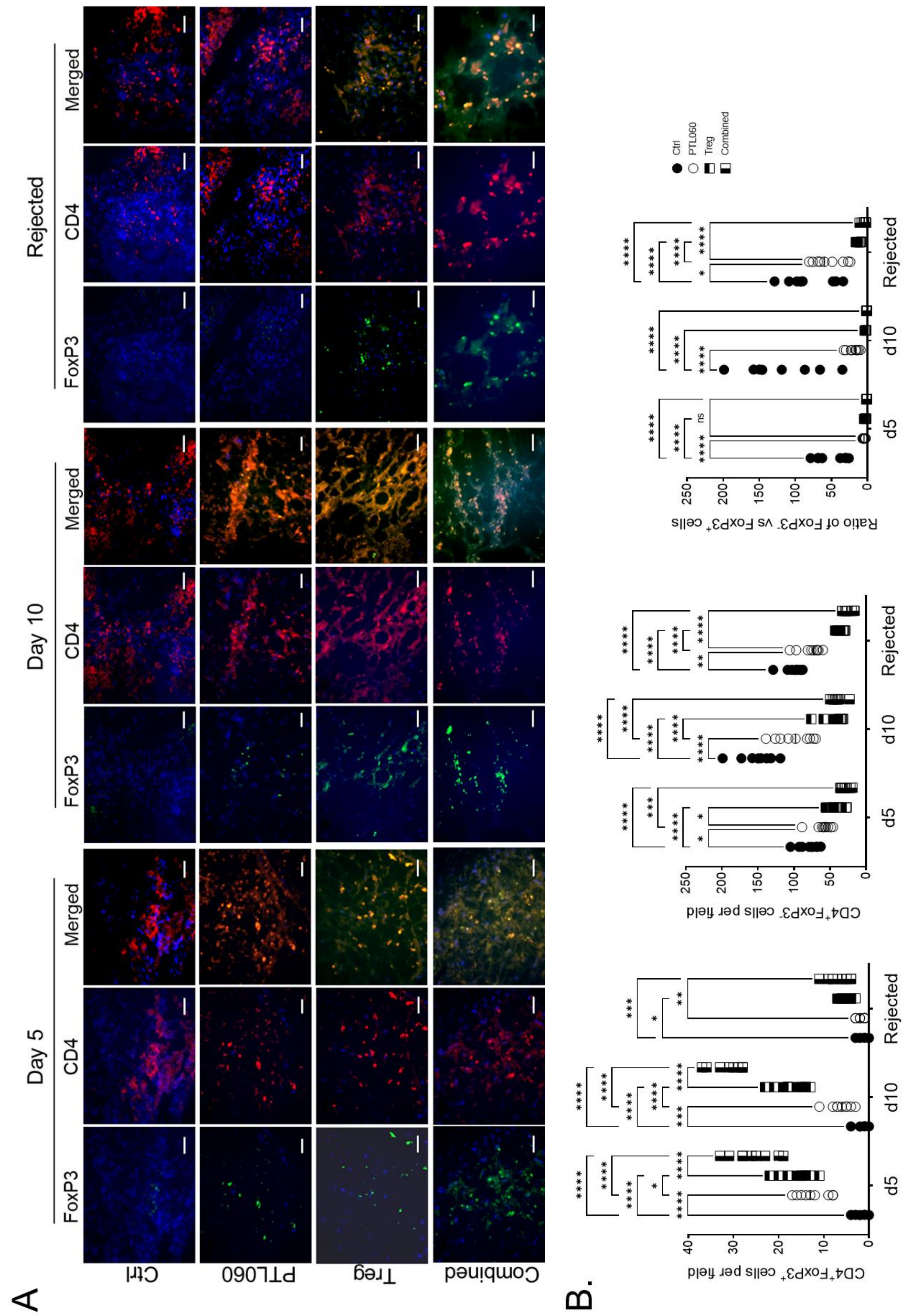

Suppl Figure 4. Analysis of CD4^{+}FoxP3^{+} and CD4^{+}FoxP3^{-} cells in heart grafts. Frozen sections of donor hearts untreated (Ctrl) or pretreated with PTL060 and from mice injected with Tregs. The hearts were harvested at day 5 or 10 after transplantation or at the time of rejection from the four groups of mice. Representative photomicrographs of OCT sections of the donor heart stained with FoxP3 (green), CD4 (Red) and DAPI for nucleus (Blue) from each treatment group (A). Cumulative graph of the number of FoxP3^{+} and FoxP3^{-} cells per field with magnification x400 of the donor heart from each group and ratio of FoxP3^{-} vs FoxP3^{+} cells in the grafts (B). Graph represents mean±SEM from three donor hearts for day 5 and from four donor hearts for day 10 or when rejected from each group. Scale bars: 25 µm. Data were analyzed by Two-way ANOVA Tukey's multiple comparisons test. ** $p < 0.01$, *** $p < 0.005$, **** $p < 0.0001$ in comparison between the four groups at day 5 or 10 or when rejected.

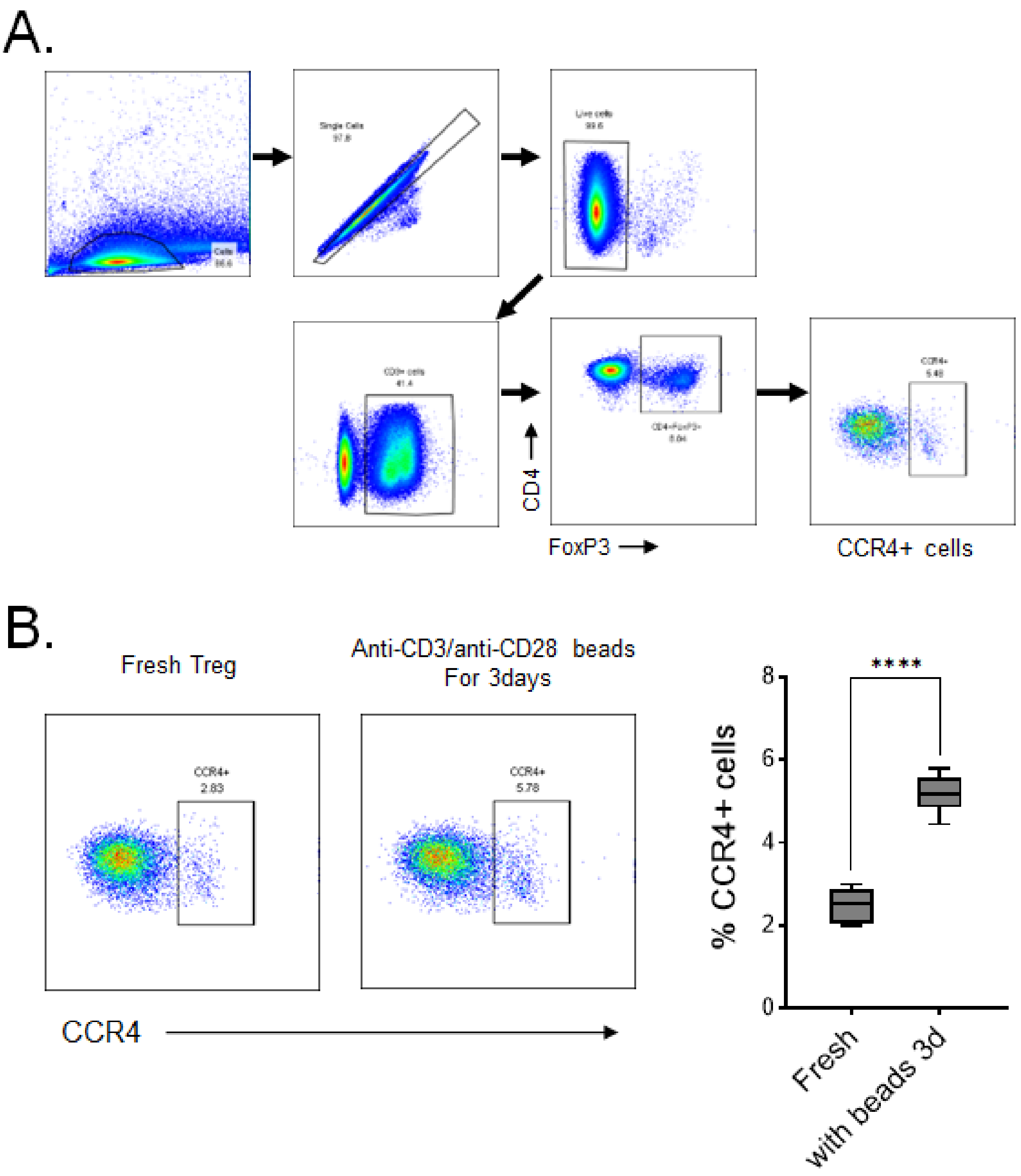

Suppl. Fig 5. Polyclonally expanded Tregs had increased expression of CCR4. The percentages of cells expressing CCR4 was assessed on the freshly isolated or 3-days expanded Tregs by flow cytometry with a gating strategy (A). The percentages of $CCR4^{+}$ Tregs were analyzed and shown in the cumulative graph (B). Dead cells excluded using near IR live/dead cell staining kit. Data were analyzed by Two-way ANOVA Tukey's multiple comparisons test. **** $p < 0.0001$ in comparison between the freshly isolated and the expanded Tregs.
